# Role of community pharmacy professionals in child health service provision in Ethiopia: a cross-sectional survey in six cities of Amhara regional state

**DOI:** 10.1186/s12913-022-08641-8

**Published:** 2022-10-18

**Authors:** Asnakew Achaw Ayele, Suzanne Cosh, Md Shahidul Islam, Leah East

**Affiliations:** 1 Department of Clinical Pharmacy, School of Pharmacy, College of Medicine and Health Science, University of Gondar, Gondar, Ethiopia; 2 School of Health, Faculty of Medicine and Health, University of New England, 2351 Armidale, Australia; 3 School of Psychology, Faculty of Medicine and Health, University of New England, 2351 Armidale, Australia; 4 School of Nursing & Midwifery, Faculty of Engineering & Science, University of Southern Queensland Toowoomba, 4305 Queensland, QLD Australia

**Keywords:** Child health, Community drug retails outlet, Community pharmacy, Community pharmacy professionals, Health service

## Abstract

**Background:**

Community pharmacy professionals have great potential to deliver various public health services aimed at improving service access, particularly in countries with a shortage of health professionals. However, little is known about their involvement in child health service provision in Ethiopia.

**Objective:**

The purpose of this study was to evaluate the level of involvement of community pharmacy professionals in child health service provision within Ethiopia.

**Methods:**

A multi-center cross-sectional survey was conducted among 238 community pharmacy professionals from March to July 2020 in Amhara regional state of Ethiopia. Independent samples t-test and one way Analysis of Variance (ANOVA) was used to test the mean difference.

**Results:**

Most community pharmacy professionals were ‘involved’ in providing child health services related to *‘advice about vitamins/supplements’* (46.6%), *‘advice about infant milk/formulas’* (47.1%) and *‘responding to minor symptoms’* (50.8%) for children. The survey revealed that, community pharmacy professionals were less frequently involved in providing childhood *‘vaccination’* services. Further, level of involvement of community pharmacy professionals differed according to participants’ licensure level, setting type, responsibility in the facility and previous training experience in child health services.

**Conclusion:**

Community pharmacy professionals have been delivering various levels of child health services, demonstrating ability and capacity in improving access to child health services in Ethiopia. However, there is a need for training and government support to optimize pharmacist engagement and contribution to service delivery.

## Background

Lowering child morality is a global priority with the United Nations (UN) Sustainable Development Goal (SDG) 3.2 calling for a reduction in under-five mortalities to at least 25/1000 live births and neonatal mortality to at least 12 per 1000 live births by 2030 [1, 2]. Despite remarkable progress having been achieved in the reduction of global under five mortalities in the past three decades, the rate of death is still high in sub-Saharan Africa and south Asian countries (4.2 million deaths as of 2019) [3]. While the two regions (sub-Saharan Africa and south Asia) alone contributed to 80% of global under five moralities in 2019, about half of these global deaths occurred in just five countries: Nigeria, India, Pakistan, Democratic Republic of the Congo, and Ethiopia [3]. The 2019 global child mortality estimation result shows that Ethiopia’s progress towards reducing newborns and under five mortality lies at 28 and 50.7 per 1000 live births, compared with 59 and 200 per 1000 live births in 1990 [3]. Although trends in declining child mortality in Ethiopia have been recorded from 1990 to 2019, the rate of both under five and infant mortality is unacceptably high and far from the SDG targets of reducing neonatal mortality to 12 deaths per 1,000 live births and under five mortalities to 25 deaths per 1,000 live births by 2030 [4].

Access to child health services remains an important instrument in lowering child mortality rates in order to achieve SDG targets. However, the significant shortage of skilled health professionals poses challenges and inequities in access to basic child health services in low-income countries [5]. The World Health Organization (WHO) suggests the integration of various health professionals in the primary healthcare system, especially in developing countries, to alleviate the shortage of skilled health professionals and to increase access to universal health coverage is needed [6]. Optimizing the roles of community pharmacy professionals in different public health settings, including child health provision could improve both access to services and minimize costs associated with hospital visit [7, 8].

Community pharmacy professionals’ role in primary health care has been increasing globally for the past two decades from being limited to dispensing medications, to involvement in direct patient care for different age groups [8–13]. When it comes to child health, community pharmacy professionals can play prominent roles in service provision, including routine immunization, advice about vitamins, management of minor ailments, counseling about infant milk formulas and chronic disease management [14–17]. Currently, in Europe community pharmacists are an important source of primary care services for children [18]. Further, some developed countries such as the United Kingdom have integrated community pharmacy into primary health care to improve access to various public health services including child health services [19, 20].

In developing countries including Ethiopia, evidence regarding the involvement of community pharmacists in child health service provision is limited. Our previously published systematic review conducted in this area identifies that there are few studies from the context of developing countries [21]. Currently, there is paucity in the literature from Ethiopia that focused on the involvement of community pharmacists in public health services in general and the evidence that is available does not include information about their engagement in child health service provisions [22]. The few studies that reported child health services were specific to management of acute diarrhea with no details related to the objectives of our study [23, 24]. Therefore, the aim of this study was to provide evidence on the extent of involvement of community pharmacy professionals in various aspects of child health service provision in Ethiopia. The findings of this study could be used as input for policy development and new initiatives concerning the role of pharmacists in child health service in community pharmacy settings in developing countries in general, and the Amhara region of Ethiopia in particular.

## Methods

### Study design, study area and sampling

This study employed a multicenter cross-sectional survey to assess the level of involvement of community pharmacy professionals in providing child health services in one regional state of Ethiopia. The study was conducted with community pharmacy professionals practicing in Community Drug Retail outlets (CDROs) of six randomly selected zonal cities (out of 11) in the Amhara regional state, Ethiopia. Selected cities were (1) Debre Markos, (2) Gondar, (3) Dessie, (4) Bahir Dar, (5) Debre Tabor and (6) Debre Birhan. The region is the second most populous region among the 11 regional states in Ethiopia with a total projected population of 22,877,365(2022) and an area of 154,709 km2 [25]. As of November 2019, there were 852 active CDROs in Amhara regional state. The total sample needed for the study was calculated using single proportion formula with the assumptions of 5% margin of error, 95% confidence level, and 50% esponse distribution. The sample calculation further employed correction formula given that the total number of sampling frames found in the study region were 852 active CDROs, which is less than 10,000. Following the correction formula, the study sample was 264 community pharmacy professionals practicing in the selected cities.

### Inclusion and exclusion criteria

Qualified and registered pharmacy professionals who had been working for at least three months in the selected CDROs were included in this study. Pharmacy professionals who had less than three months of work experience in CDROs were excluded to reduce under exposure bias, assuming that they may not have had adequate exposure to child health services. Intern pharmacy students were also excluded due to their limited practice.

### Data collection tools and procedures

A self-administered questionnaire was developed by the research team from the published literature relevant to the potential role of pharmacists in child health service provision [26–28]. The questionnaire was further reviewed by three experts in pharmacy practice and public health to ensure content validity. Content of the questionnaire was modified according to feedback from the experts. A pilot study was conducted with 10 community pharmacy professionals recruited from two study sites prior to data collection to check the clarity of the survey. Following the pilot study, minor changes in wordings and phrasing were incorporated. There were two parts of the questionnaire: the first part assessed information related to the study participants’ characteristics such as gender, educational achievements, professional licensing level, experience in the CDROs, responsibility in the CDROs, and training experience regarding child health services. The second part of the questionnaire was designed to assess involvement of community pharmacy professionals in child health service provision. The second part of the questionnaire had four items and responses to these questions were recorded on four-point Likert-type scales as *‘very involved’, ‘involved’, ‘little involved’ and ‘not all involved’* with scores of 4,3,2 and 1 respectively. By summing responses to the four items, the maximum score is 16 and the minimum score is four. Higher total scores indicate higher involvement in the provision of child health services. The items pertaining to involvement in service provision demonstrated adequate internal consistency α = 0.775.

Random sampling technique was employed to recruit all study participants. Lists of currently functional CDROs in the study sites (selected zonal cities) were requested and accessed from zonal administration health departments. Then the proportions of CDROs needed in each city were calculated considering the total sample size. Equal proportion was not given because the number of CDROs were not equal in each city. From the accessed lists, samples of CDROs were randomly selected to recruit study participants (one pharmacy professional/CDRO). Trained data collectors (pharmacists) recruited study participants randomly from the list of selected CDROs at each study site. Once the data collectors identified the licensed pharmacy professionals in the CDRO, they provided participant information and consent forms. The self-reported survey questionnaires were provided to participants who signed the consent form. Data collection was undertaken between March and July 2020.

### Data analysis

The data were analyzed using the Statistical Package for the Social Sciences (SPSS), version 26 (SPSS, Inc., Chicago, IL, USA). While frequencies and percentages were used to summarize categorical variables, means with standard deviations were used to summarize continuous variables. The total involvement score was computed as a sum of the responses scored in each item to construct an approximately continuous variable. Sum of responses of all the ordinal Likert scales in each item is much higher than the scores of the individual items which could result in an approximately continuous variable. Independent sample t-test (for two group variables) and one way Analysis of Variance (ANOVA) was employed to test the mean difference of involvement scores of community pharmacy professionals in child health service provision among subgroups of participant characteristics. Where ANOVAs were significant, Bonferroni post analyses were used to identify a further mean difference. Level of statistical significance was determined at a two-sided p-value < 0.05.

## Results

### Characteristics of the study participants

From the total of 264 community pharmacy professionals approached, 238 participants completed the questionnaires: a response rate of 90%. Characteristics of the study participants are presented in Table 1.

**Table 1:** Characteristics of the community pharmacy professionals included in the study

Characteristics	Total (n = 238)
Characteristics of the community pharmacy professionals	N (%)
**Sex**	
Male	117(49.2)
Female	121(50.8)
**Educational qualification in Pharmacy**	
Diploma in Pharmacy	130(54.6)
Bachelor of Pharmacy (BPharm)	91(38.2)
Master of Pharmacy (MSc)	17(7.1)
**Work experience in years (Mean, SD)**	6.05 ± 5.49
Less than 5 years	130(54.6)
5–10 years	40(16.8)
Greater than 10 years	68(28.6)
**Licensure by regulatory Authority**	
Druggist/Pharmacy technician	96(40.3)
Junior Pharmacist	48(20.2)
Senior Pharmacist/Druggist	45(18.9)
Chief Pharmacist	19(8.0)
Expert Pharmacist	30(12.6)
**Facility (CDRO)type**	
Drug store	106(44.5)
Pharmacy	132(55.5)
**Responsibility in the CDRO**	
Owner	96(40.3)
Employed	142(59.7)
**Have you received any in -services training regarding maternal and child health services delivery in CDROs?**	
Yes	42(17.6)
No	196(82.4)

### Involvement of community pharmacy professionals in child health services provision

As presented in Table 2, community pharmacy professionals had the highest mean involvement score in ‘advising about vitamins/ supplements’ to children (mean = 3.24, SD = 0.717). The percentage distribution also showed that most community pharmacy professionals reported that they have been either ‘very involved’ (39.5%) or ‘involved’ (46.6%) in ‘advising about vitamins/ supplements’ to children. The second highest mean involvement score of community pharmacy professionals was in providing child health services related to ‘advising about infant formulas /milk’, with 37% ‘very involved’ and 47.1% ‘involved’. Most community pharmacy professionals also reported involvement in child health service provision related to ‘responding to minor symptoms’, where more than 50% of the participants reported being ‘involved’. The lowest mean involvement score was provision of child health service related to ‘vaccination’ (mean = 2.65, SD = 0.923). Compared to the other child health services, a higher proportion of participants were a ‘little involved’ (30.7%) and ‘not at all involved’ (11.8%) in providing services related to vaccinations. Additional details are provided in Table 2.

**Table 2:** Percentage distribution and mean levels of involvement of community pharmacy professionals in child health services

Child health service	Very involved, n (%)	Involved, n (%)	Little involved n (%)	Not at all involved, n (%)	Mean involvement score	Std. Deviation
Advising about vitamins/ supplements	94(39.5)	111(46.6)	30(12.6)	3(1.3)	3.24	0.717
Provision of vaccination/ Checking the child’s vaccination status and refer for vaccination/ provide child vaccination advice to the parent	46(19.3)	91(38.2)	73(30.7)	28(11.8)	2.65	0.923
Responding to minor symptoms (fever, acute diarrhea, respiratory symptoms)	79(33.2)	121(50.8)	32(13.4)	6(2.5)	3.15	0.740
Advising about infant formulas /milk	88(37.0)	112(47.1)	30(12.6)	8(3.4)	3.18	0.776
Overall mean involvement score					12.22	2.452

### Difference in the involvement of community pharmacy professionals in child health provision based on sub-groups of respondents’ characteristics

As depicted in Table 3, significant mean difference of involvement in child health service provision was observed among different sub-groups of pharmacists. To be more specific, significant differences in mean involvement was observed based on educational qualifications in Pharmacy, professional licensure levels, Facility (CDRO)types, responsibilities in the CDROs and in services training status. A significantly higher level of pharmacy professionals’ involvement in child health service provision was observed among those working in ‘pharmacy’ settings compared to those working in ‘drug store’ settings. Involvement of community pharmacy professionals in child health service provision was also significantly higher among CDRO ‘owners’ in comparison to their counterparts (‘employees’). Additionally, a significant difference in involvement was also observed based on ‘in-service training status’, with less involvement in child health service provision observed among those who did not report having received training about child health service provision compared to those who had received training. The post-hoc analyses (see Table 4) showed that significant differences in mean involvement score was observed among sub-groups of the study participants based on professional ‘licensure level’. Involvement scores in child health services provision were higher among community pharmacy professionals with licensure level of ‘senior pharmacist’ and ‘expert pharmacist’ compared to pharmacy professionals with a licensure level of ‘druggist/ pharmacy technicians’.

**Table 3:** Total involvement of community pharmacy professionals in provision of child health service among different subgroups of respondents’: Independent sample t-test and one way ANOVA

Demographic and related characteristics	Categories	Involvement in child health service		
		**Mean ± SD**	**P-value- mean difference**	**P-value- Levene’s Test for Equality of Variances**
**Sex**	Male	12.38 ± 2.480	0.331	0.557
	Female	12.07 ± 2.425		
**Educational qualification in Pharmacy**	Diploma in Pharmacy	11.85 ± 2.594	0.034*	0.450
	Bachelor of Pharmacy (BPharm)	12.59 ± 2.196		
	Master of Pharmacy (MSc)	13.00 ± 2.264		
**Work experience in years**	Less than 5 years	11.89 ± 2.428	0.074	0.701
	5–10 years	12.67 ± 2.506		
	Greater than 10 years	12.50 ± 2.311		
**Licensure by regulatory Authority**	Druggist/Pharmacy technician	11.39 ± 2.114	< 0.001*	0.124
	Junior Pharmacist	12.38 ± 2.878		
	Senior Pharmacist/Druggist	13.16 ± 2.256		
	Chief Pharmacist	12.79 ± 2.440		
	Expert Pharmacist	12.87 ± 2.300		
**Facility (CDRO)type**	Drug store	11.72 ± 2.544	0.004*	0.973
	Pharmacy	12.62 ± 2.307		
**Responsibility in the CDROs**	Owner	12.79 ± 2.190	0.003*	0.158
	Employed	11.83 ± 2.549		
**In -services training received**	Yes	13.38 ± 2.337	0.001*	0.868
	No	11.97 ± 2.409		

*The mean difference is significant at the 0.05 level

**Table 4:** Post hoc analyses of factors influencing community pharmacy pharmacists’ involvement in child health services

Variables	Factors	Group	Mean difference	P-value	95% Confidence Interval	
					**Lower Bound**	**Lower Bound**
Educational qualification in Pharmacy	Diploma in pharmacy	Bachelor of Pharmacy (BPharm)	− 0.740	0.080	-1.54	0.06
		Master of Pharmacy (MSc)	-1.146	0.205	-2.66	0.36
	Bachelor of Pharmacy (BPharm)	Diploma in pharmacy	0.740	0.080	− 0.06	1.54
		Master of Pharmacy (MSc)	− 0.407	1.000	-1.95	1.14
	Master of Pharmacy (MSc)	Diploma in pharmacy	1.146	0.205	− 0.36	2.66
		Bachelor of Pharmacy (BPharm)	0.407	1.000	-1.14	1.95
Licensure by regulatory Authority	Druggist/Pharmacy technician	Junior Pharmacist	− 0.990	0.185	-2.17	0.19
		Senior Pharmacist	-1.770*	< 0.001	-2.98	− 0.56
		Chief Pharmacist	-1.404	0.187	-3.08	0.28
		Expert Pharmacist	-1.481*	***0.030***	-2.88	− 0.08
	Junior Pharmacist	Druggist/Pharmacy technician	0.990	0.185	− 0.19	2.17
		Senior Pharmacist	− 0.781	1.000	-2.17	0.61
		Chief Pharmacist	− 0.414	1.000	-2.23	1.40
		Expert Pharmacist	− 0.492	1.000	-2.05	1.07
	Senior Pharmacist	Druggist/Pharmacy technician	1.770*	< 0.001	0.56	2.98
		Junior Pharmacist	0.781	1.000	− 0.61	2.17
		Chief Pharmacist	0.366	1.000	-1.46	2.20
		Expert Pharmacist	0.289	1.000	-1.29	1.87
	Chief Pharmacist	Druggist/Pharmacy technician	1.404	0.187	− 0.28	3.08
		Junior Pharmacist	0.414	1.000	-1.40	2.23
		Senior Pharmacist	− 0.366	1.000	-2.20	1.46
		Expert Pharmacist	− 0.077	1.000	-2.04	1.88
	Expert Pharmacist	Druggist/Pharmacy technician	1.481*	0.030	0.08	2.88
		Junior Pharmacist	0.492	1.000	-1.07	2.05
		Senior Pharmacist	− 0.289	1.000	-1.87	1.29
		Chief Pharmacist	0.077	1.000	-1.88	2.04

* The mean difference is significant at the 0.05 level

## Discussion

This study highlights the involvement of community pharmacy professionals in providing child health services in Ethiopia. Overall, this study showed that, community pharmacy professionals in Ethiopia have been involved in providing different types of child health services. To be more specific, advice about *‘vitamins/ supplements’, advice about ‘infant formula/milk’ and ‘responding to minor symptoms’* were the child health services most frequently provided by the community pharmacy professionals surveyed. This indicates the growth of community pharmacy professionals’ role and involvement in providing various child health services in general in Ethiopia. Published evidence has also reported the increase in utilization of community pharmacy-based child health service delivery particularly in relation to immunization services, responding to minor ailments and management of chronic disease [14, 16, 18, 29]. Engaging community pharmacy professionals in child health service provision could reduce service demands in clinical settings, particularly in resource limited countries including Ethiopia. For example, managing minor symptoms in children in community pharmacy settings could improve access to the service and reduce costs related to unnecessary hospital visits [7]. In addition, the high involvement of community pharmacy professionals in providing advice about *‘vitamins/ supplements’* and ‘infant formula/milk’ have the potential to increase awareness about fraudulent products among clients. Evidence suggests that fraudulent food products are highly prevalent in developing countries [30]. In tackling these problems, enhancing services of community pharmacy professionals in providing advice about *‘vitamins/ supplements’ and ‘infant formula/milk’* could be an imperative strategy considering their knowledge and professional skills.

Although community pharmacy professionals reported that they have been involved in child health service provision, their reported involvement in certain services such as vaccinations was limited. The low level of involvement in providing childhood vaccination services might be due to lack of policy support by the ministry of health of Ethiopia. This is because in most cases, childhood vaccination services have been delivered in public health facilities. In developing countries including Ethiopia, childhood vaccination uptake is challenging and low in coverage [31]. In such cases, considering their wider availability and long opening hours, enabling community pharmacy professionals to provide vaccination services might be helpful to improve uptake and access [32]. Unlike Ethiopia, there are countries who have authorized community pharmacy professionals as providers of various types of vaccinations for different age groups including children [33–36].

The extent of involvement in providing child health services was found to be varied based on community pharmacy professionals’ professional licensure levels, facility types, responsibilities in the CDROs and training status. For example, higher level of involvement was observed among pharmacy professionals who had been working in ‘pharmacy’ settings compared with in a ‘drug store’. The low level of involvement in the drug stores could be associated with regulatory authorizations. In Ethiopia, drug stores are authorized to sell limited types of medications which might limit their future involvement in providing child health services, particularly medication related advice. Similarly, licensure level in pharmacy was found to be linked with variation in level of involvement in overall child health service provision. The low level of involvement in child health service provision among pharmacy professionals with licensure level ‘druggist/ pharmacy technician’ could be linked with the limited exposure to courses relevant to child health during their formal pharmacy education. This is because both the courses and duration of training for pharmacy technicians are less than a bachelor’s degree in pharmacy. However, through additional training and capacity building, pharmacy professionals with lower educational attainment could be empowered to engage in providing child health services. The effectiveness of educational interventions and professional training to improve knowledge and practice skills of pharmacy professionals have been evidenced in the published literature [37, 38].

Moreover, involvement of community pharmacy professionals in child health service provision was also significantly higher among CDRO ‘owners’ compared with ‘employees’. This can be seen from two perspectives: business motivated involvement and the freedom of choices for action. From the first perspective, the pharmacy owner might not hesitate to provide services thinking of the business he/she can make. The second perspective is related with freedom of practice, where employees might not have full freedom of providing what services.

## Strengths and limitations of the study

We acknowledge that this study has limitations to be considered while interpreting the results. Firstly, the cross-sectional nature of the study design cannot show trends of changes over time among community pharmacy professionals’ involvement in child health service delivery. Secondly, the findings are based on self-reported responses which is susceptible to social desirability bias. However, despite these limitations, the data in this study were generated from multiple centers that employed appropriate sampling techniques in order to fill evidence gaps in the area. Although this study was conducted in one region (Amhara regional state), the findings could be representative to other regions of the country considering the similarity in pharmacy professionals and CDROs regulations. This is because issues related with health professionals and health facilities including pharmacists and CDROs in Ethiopia are mainly regulated centrally by Ethiopian food and drug authority.

## Implications for policy, future practice, and research

There are no clear directives or policy that supports the expanded role of community pharmacy professionals in providing different public health services including child health services in Ethiopia. Therefore, taking the potential role of pharmacy professionals in child health delivery into consideration, development of national policy that clearly defines how pharmacy professionals could engage in providing various types of child health services in community pharmacy settings in Ethiopia is needed. In addition, to maximize the benefits of community pharmacy-based child health service delivery, providing capacity building training and empowerment is needed to address the potential gaps in practice. The findings of this study have provided some insights about the potential role of community pharmacy professionals in child health service delivery in Ethiopia and developing countries in general. Exploring the barriers and facilitators to community pharmacy professional in providing child health services could be an area of future research.

## Conclusion

Community pharmacy professionals have been involved in providing child health services such as advice about vitamins/supplements, advice about infant milk/formulas and responding to minor symptoms for children. The increasing role of pharmacy professionals beyond merely dispensing of medications has the potential to improve access to child health services. Integrating and enhancing the role of community pharmacists in child health care service delivery could assist countries with their efforts in reducing child mortality and, subsequently, in meeting SDG targets. Empowering community pharmacy professionals through training focusing on child health service provision would also be needed to improve their involvement in the service delivery.

## Data Availability

All relevant data are included in this manuscript. Data may be shared upon reasonable request is provided to the corresponding author.

## List of abbreviations

ANOVA: Analysis of Variance
CDROs: Community Drug Retail outlets
SDG: Sustainable Development Goals
UN: United Nations
WHO: World Health Organization
